# Co-profiling reveals distinct patterns of genomic chromatin accessibility and gene expression in pulmonary hypertension caused by chronic hypoxia

**DOI:** 10.1186/s12931-023-02389-3

**Published:** 2023-04-08

**Authors:** Dongdong Yu, Ting Zhang, Guangyuan Zhou, Zeang Wu, Rui Xiao, Han Zhang, Bingxun Liu, Xiangpan Li, Matthieu Ruiz, Jocelyn Dupuis, Liping Zhu, Qinghua Hu

**Affiliations:** 1 Department of Pathophysiology, School of Basic Medicine, Wuhan, China; 2 Key Laboratory of Pulmonary Diseases of Ministry of Health, Tongji Medical College, Huazhong University of Science and Technology, Wuhan, 430030 China; 3 Department of Oncology, Renmin Hospital, Wuhan University, Wuhan, China; 4 Department of Nutrition, Université de Montréal, Montreal, Canada; 5 Montreal Heart Institute, Montreal, QC Canada; 6 Department of Medicine, Université de Montréal, Montreal, QC Canada

**Keywords:** Pulmonary hypertension, Chromatin accessibility, PASMC, DAR, DEG

## Abstract

**Introduction:**

Aberrant gene expression is a key mechanism underlying pulmonary hypertension (PH) development. The alterations of genomic chromatin accessibility and their relationship with the aberrant gene expressions in PH are poorly understood. We used bulk Assay for Transposase-Accessible Chromatin with high-throughput sequencing (ATAC-seq) and RNA sequencing (RNA-seq) in pulmonary artery smooth muscle cells (PASMCs) of chronic hypoxia-exposed rats mimicking group 3 human PH.

**Methods:**

Adult Sprague Dawley rats were commercially obtained from Hunan SJA (Hunan SJA Laboratory Animal Co., Changsha, China) and randomizedly allocated into four groups exposing to nomobaric hypoxia or normoxia for 1 or 28 days respectively. After the assessment of pulmonary hemodynamics, smooth muscle cells were isolated from intralobular arteries and simultaneously subjected to bulk Assay of ATAC-seq and RNA-seq.

**Results:**

Hypoxic exposure for continuous 28-days, but not for 1-day, induced established PH phenotypes in rats. ATAC-seq revealed a major distribution of differential accessibility regions (DARs) annotated to the genome in out-of-promoter regions, following 1-day or 28-days hypoxia. 1188 DAR-associated genes and 378 differentially expressed genes (DEGs) were identified in rats after exposure to 1-day hypoxia, while 238 DAR-associated genes and 452 DEGs for 28-days hypoxia. Most of the DAR-associated genes or DEGs in 1-day did not overlap with that of 28-days hypoxia. A Pearson correlation analysis indicated no significant correlation between ATAC-seq and RNA-seq.

**Conclusions:**

The alterations in genomic chromatin accessibility and genes expression of PASMCs in the initial stage of hypoxia are distinct from the established stage of hypoxia-induced PH. The genomic differential accessibility regions may not be the main mechanisms directly underlying the differentially expressed genes observed either in the initial or established stages of PH. Thus the time-course alterations of gene expression and their possible indirect link with genomic chromatin accessibility warrant more attention in mechanistic study of pulmonary hypertension.

## Introduction

Pulmonary hypertension (PH) is a clinical syndrome associated with progressive rise in pulmonary vascular resistance resulting in right heart failure and high lethality. Aberrant gene expression, a key mechanism underlying PH development [1], results from changes in chromatin accessibility, transcriptional promoter regulation and post-transcriptional modifications. However, the alterations of genomic chromatin accessibility and their relationship with the aberrant gene expressions in PH are poorly understood. We therefore used bulk Assay for Transposase-Accessible Chromatin with high-throughput sequencing (ATAC-seq) and RNA sequencing (RNA-seq) in pulmonary artery smooth muscle cells (PASMCs) of chronic hypoxia-exposed rats mimicking group 3 human PH.

## Methods

Twenty four adult, male Sprague Dawley rats, weight at 320–370 g, were randomizedly assigned into four groups either exposing to normobaric hypoxia of 10% oxygen balanced by nitrogen gas with air, or to normobaric normoxia with air for 1 or 28 days respectively. The rats were housed in chambers with the above hypoxic and normoxic condition continuously except for chamber cleaning and had free access to food and water. After assessment of pulmonary hemodynamics and Fulton’s index of right ventricle as we previously described in full [2], smooth muscle cells were carefully removed mechanically from media of intralobular arteries [2, 3] and quickly prepared for the subsequent nuclei and RNA isolation and the final bulk assay of ATAC-seq and RNA-seq.

## Results

**Fig. 1:**
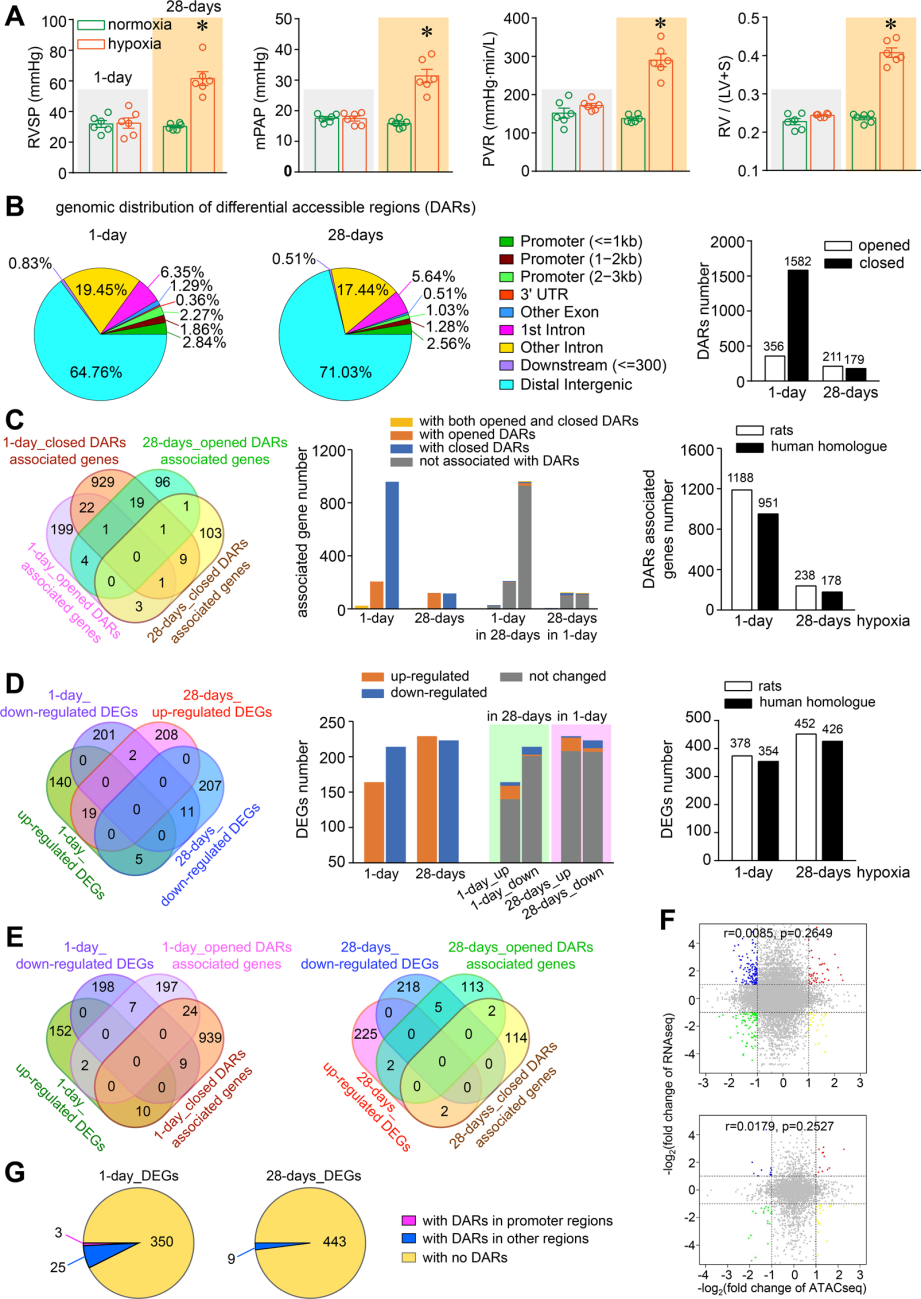
**A** Pulmonary hypertension phenotype assessment in SD rats. Adult male Sprague Dawley rats (320–370 g) were randomly assigned into air or hypoxic chamber with 10% oxygen for 1 or 28 days. After pulmonary hemodynamic assessment for right ventricular systolic pressure (RVSP), mean pulmonary artery pressure (mPAP), pulmonary vascular resistance (PVR) and right ventricle hypertrophy [right ventricular weight/left ventricular plus septal weight, RV/(LV + S)], n = 6 for each, *P < 0.05. Pulmonary artery smooth muscle cells (PASMCs) were mechanically removed carefully from intra-lobe pulmonary arteries and subjected to ATAC-seq and RNA-seq. **B** Genomic distribution of differential accessibility regions (DARs). **C** Comparison of genes annotated with DARs including opened or closed chromatin accessibility (*left* and *middle*) and homologues with human (*right*). **D** Comparison of differentially expressed genes (DEGs) including up-regulated and down-regulated genes (*left* and *middle*) and homologues with human (*right*). **E** Comparison of DARs and DEGs. **F** Pearson correlation analysis of RNA-seq and ATAC-seq in PASMCs of rats after exposure to hypoxia for 1- (*upper*) or 28-days (*lower*), fold change > 2, *P* > 0.05 as compared to normoxia. **G** Comparison of DEGs and DARs in promoter region. All in PASMCs of rats after exposure to hypoxia for 1- or 28-days, fold change > 2, *P* < 0.05 as compared to normoxia (**B, C, D, E** and **G**)

Hypoxic exposure for continuous 28-days, but not for 1-day, induced established PH phenotypes in rats (Fig. 1A). Following 1-day hypoxia, ATAC-seq revealed a major distribution of differential accessibility regions (DARs, fold change > 2 and *P* < 0.05) annotated to the genome in out-of-promoter regions such as the distal intergenic regions (64.76%) or other intronic regions (19.45%), but only < 7% in promoter regions (Fig. 1B). In addition, DARs distribution pattern following 28-days hypoxia was similar to 1-day, with < 5% in promoter regions (Fig. 1B).

When the nearest genes or regulatory elements were annotated to the above DARs, 1188 (982 closed- and 230 open-DARs including 24 with both open and closed) or 238 (122 open- and 118 closed-DARs including 2 with both open and closed-) DAR-associated genes were found for 1- or 28-days hypoxia (Fig. 1C). RNA-seq identified 378 (214 down- and 164 up-regulated) and 452 (229 up- and 223 down-regulated) differentially expressed genes (DEGs) for 1- or 28-days hypoxia (Fig. 1D). The DAR-associated genes and DEGs revealed in rat models are in 74.79–80.05 or 93.65–94.25% homology with human (Fig. 1C, D), indicating the high potential relevance of this experimental model in comparison with human PH. We compared the human homologous of the DEGs with the human hypoxia-related genes obtained from the HALLMARK_HYPOXIA gene set in the MSigDB database [4] (www.gsea-msigdb.org/gsea/msigdb/human/geneset/HALLMARK_HYPOXIA.html). There are 12 and 11 overlapping genes in 1-day and 28-days hypoxia, and 9 of them have been documented in association with pulmonary hypertension in literatures including Bhlhe40, Cdkn1a, Maff, Tnfaip3, Atf3, Pim1, Serpine1, Irs2 and Selenbp1.

Most of the DAR-associated genes or DEGs in 1-day did not overlap with that of 28-days hypoxia (Fig. 1C, D), demonstrating that the genes expression reprogrammings in the initial stage is different from the ones responsible for the final establishment and/or maintenance of PH phenotypes and vice versa.

Only 28 out of the total 378 and 9 out of 452 altered genes (DEGs) were accompanied with changed chromatin accessibility (DAR-associated genes) at 1- and 28-days hypoxia respectively (Fig. 1E). A Pearson correlation analysis indicated no correlation between ATAC-seq and RNA-seq (fold change, *P* > 0.05, Fig. 1F). The absence of correlation may suggest that DARs may not be the main mechanisms directly underlying DEGs observed either in the initial or established stages of PH.

Furthermore, only 3 out of a total 830 DEGs comprising 378 in 1-day and 452 in 28-days hypoxia were associated with DARs changes in promoter regions (Fig. 1G), indicating that almost none of the above changes in genes expressions directly resulted from alterations in local chromatin accessibility or promoter activity.

## Discussion

The very low and similar distribution of DARs in the promoter region (Fig. 1B) indicates that alterations in regions out of promoters, not just transcriptional promoters alterations as already known may also be pathophysiologically important for either the initial or established stage of PH. Although to be validated (Fig. 1C, D), we can speculate that the aberrant gene expression in the initial stage may pave the way for the initial pulmonary remodeling that will in turn mediate the second step of gene expressions reprogramming eventually responsible for the onset and maintenance of PH. Different alterations between 1-day and 28-days emphasize the importance of time-based analysis in the interpretation of pathophysiology studies and therapeutic strategies investigations using the acute or chronic hypoxia in animal models. In particular, time window of therapeutic interventions in relation with genomic alterations could have a great impact on results and benefits.

Hypoxia-induced chromatin accessibility and gene expression changes were not concordant in PASMCs (Fig. 1E–G). Similarly, a study also found that local chromatin accessibility and transcription were not concordant in MCF-7 breast cancer cells [5]. Finally, another study on chromatin accessibility of primary human cancers showed that only 24% of ATAC-seq peaks correlated with nearest genes [6].

Considering more than 80% distribution of DARs in the distal intergenic regions or other intronic regions (Fig. 1B), non-coding transcripts from these regions in the genome can be a relevant alterative mechanism underlying aberrant gene expressions. The non-coding transcripts can regulate transcriptional gene expression or modulate mRNA stability [7]. An additional post-transcriptional regulation includes the RNA-binding proteins in mastering gene expression profiles [8].

## Conclusion

The alterations in genomic chromatin accessibility and genes expression of PASMCs in the initial stage of hypoxia are distinct from the established stage of hypoxia-induced PH. The non-coding transcripts from distal areas other than local genome loci and the post-transcriptional regulation may also play important pathophysiological roles in hypoxic PH. All these warrant more attention in future investigations to better understand both initiation and progression of PH.

## Data Availability

The datasets used and/or analyzed during the current study are available from the corresponding author on reasonable request.

## Abbreviations

PH: Pulmonary hypertension
ATAC-seq: Assay for transposase-accessible chromatin with high-throughput sequencing
RNA-seq: RNA sequencing
PASMCs: Pulmonary artery smooth muscle cells
DARs: Differential accessibility regions
DEGs: Differentially expressed genes
